# Complete Genome Sequence of *Paenibacillus* sp. Strain 37, a Plant Growth-Promoting Bacterium Isolated from the Rhizosphere of Abies nordmanniana (Nordmann Fir)

**DOI:** 10.1128/MRA.01139-19

**Published:** 2021-02-25

**Authors:** Adriana M. Garcia-Lemos, Rosanna C. Hennessy, Tue K. Nielsen, Lars H. Hansen, Mette H. Nicolaisen

**Affiliations:** a Department of Plant and Environmental Sciences, University of Copenhagen, Frederiksberg, Denmark; University of Maryland School of Medicine

## Abstract

We report the complete genome sequence of *Paenibacillus* sp. strain 37, a plant growth-promoting bacterium (PGPB) isolated from the rhizosphere of Abies nordmanniana (Stev.) Spach; it contains a single chromosome of 7.08 Mbp and one plasmid of 54.33 kbp, including 6,445 protein-coding genes, 107 tRNAs, and 13 rRNA loci.

## ANNOUNCEMENT

*Paenibacillus* spp. comprise diverse bacteria, including plant growth-promoting bacteria with potential applications in the sustainable management of plant diseases (1). We report the complete genome sequence of *Paenibacillus* sp. strain 37, previously isolated from the rhizosphere of Abies nordmanniana (Nordmann fir) seedlings sampled at Baumschule Engler, Hohenlockstedt, Germany (53°58′30.5″N, 9°37′51.9″E) (2). A single colony of strain 37 was inoculated in lysogeny broth (LB) overnight at 28°C, and genomic DNA (gDNA) was purified using the NucleoSpin kit (Macherey-Nagel, DE) according to the manufacturer’s instructions.

Sequencing was performed using the BGISEQ-500 and PacBio Sequel systems at BGI Hong Kong. For BGISEQ-500 sequencing, 1 μg genomic DNA was randomly fragmented on a Covaris instrument (Brighton, UK). DNA fragments between 200 and 400 bp were selected by Agencourt AMPure XP medium kit. The size-selected DNA fragments were end repaired and 3′ adenylated. Adaptors were ligated to the 3′-adenylated fragment ends. The DNA fragments, now including adaptors, were amplified and purified, and the double-stranded PCR products were heat denatured and circularized by the splint oligonucleotide sequence. The resulting circular DNA library was sequenced on the BGISEQ-500 sequencing platform according to the manufacturer’s protocol (3). Adapter sequences and low-quality bases (<Q20) were trimmed from the raw reads using SOAPnuke v1.5.5 software at BGI Hong Kong. PacBio sequencing used 8 μg gDNA fragmented to 10 kb with Covaris g-TUBEs. The DNA fragments were repaired to obtain blunt ends, ligated, and treated with ExoIII and ExoVII to remove linear fragments after pooling, and BluePippin was used for size selection prior to SMRTbell sequencing, followed by the removal of adapter sequences according to the PacBio Sequel system protocol at BGI Hong Kong.

A total of 6,955,122 clean reads were obtained from the BGISEQ-500 run and 134,135 reads from the PacBio run, with an average read length of 21.4 kbp. A hybrid assembly with 2 × 150-bp paired-end reads from BGISEQ-500 and long PacBio reads was generated using the Unicycler assembler v0.4.8-beta (4). The complete genome sequence was annotated using PROKKA (5) and predicted to have 6,553 genes (6,445 protein-coding genes, 13 rRNA loci, 107 tRNAs). Default parameters were used for all software unless otherwise noted.

Consensus assembly generated two contigs of 7,618,936 bp, which includes a single circular chromosome (7,075,616 bp) with a G+C content of 45.6% and a circular, single-copy megaplasmid (543,320 bp). Sequencing coverage was 137× for the BGISEQ-500 data and 380× for the PacBio data, and mean analysis of the 16S rRNA gene showed the highest sequence identity to the 16S rRNA genes of Paenibacillus tundrae A10b (99.27%; GenBank accession number NR_044525) and P. amylolyticus JCM 9906 (98.20%; NR_112163) following a search against the Greengenes nucleotide database using the Basic Local Alignment Search Tool (BLAST).

Genome mining using antiSMASH v5.0. (6) and RAST (7) revealed multiple genes associated with plant growth promotion, including siderophore biosynthesis- and phytohormone-associated genes. AntiSMASH v5.0 predicted 16 putative secondary metabolite gene clusters for synthesis of antimicrobial compounds, including paeninodin, bacitracin, paenilipoheptin, xenocoumacin, pellasoren, and octapeptin. The complete genome sequence of this strain will support future studies to determine the mechanisms underpinning the plant growth-promoting activity of this isolate and expand the genomic information currently available on plant growth-promoting bacteria.

### Data availability.

The complete *Paenibacillus* sp. strain 37 genome sequence has been submitted to GenBank under accession numbers CP043761 and CP043762. The raw sequence reads are available under BioProject accession number PRJNA563815. The SRA accession numbers for the BGISEQ (BGISEQ-500) run and for the PACBIO_SMRT (Sequel) run are SRX6811661 and SRX6811662, respectively.
